# Modern Aspects of Pain in Medicine

**Published:** 1893-10-28

**Authors:** 


### Modern Aspects of Pain in Medicine.

Probably there is no symptom for which a patient more frequently seeks advice than pain, and certainly to attain a proper understanding of its meaning and its value is a life-lasting study even for the most acute observers. It has long been known that very often pain associated with disease of an organ is not situated over that organ; for instance, pain due to disease of the liver is commonly felt in the right shoulder; a patient with disease of his heart complains of pain down the left arm; errors of refraction give rise to headache; the pain of toothache may be felt almost anywhere; the pain due to pleurisy may be on the opposite side to the inflamed pleura; pain due to disease of the stomach is often felt at the back between the shoulders, or at the junction of the first and second pieces of the sternum; that due to disease of the kidney and ureter is felt in the testicle, and down the thigh; disease of the hip causes pain in the knee; and the pain met with in diseases of the pelvic organs, both male and female, is often felt at some distant part. These facts are so well known that students and doctors are prone to forget their strangeness, and also that there must be some deep meaning in them. It has been reserved for Dr. Head to throw great light on them, and to write one of the most important papers of modern medicine. It was read before the Neurological Society last winter, and appears in the last number of *Brain*.

Several observers have from time to time pointed out that often in visceral disease certain areas of the skin could be found to be very tender; for instance, Peter has indicated that in some cases of heart disease tender spots may be found in various positions on the chest wall; and in his recently published "Manual of Medical Treatment" Dr. Burney Yeo gives the position of these tender spots in some cases observed by him. Mackenzie has also done much work on this subject. Head begins his paper by investigating these tender areas. The best "method of marking out such an area is to take a pin with a round head of such a size that it is obviously blunt to all parts of one's own cheek and face. Travel over the surface, using the blunt end only, in the same way as if you were using the point to test for analgesia. . . . The patient does not complain until the limits of the tender areas are reached, when he at once complains that he is intensely sore. . . . The touch of the head of the pin feels to him like a prick on a normal skin surface, whilst the point gives him acute pain far in excess of that caused by a prick upon the normal skin. . . . Another characteristic of these tender areas is the increased reflexes which can be obtained from them." Now, when any viscus is diseased the cutaneous area of tenderness is always the same for that viscus, and the first instance Head takes is the tenderness met with in that form of gastric disease, in which the patient suffers from pain after food, with or without hæmatemesis. In this disease there is an area of tenderness bounded above by a line extending from a point two and a half inches above the umbilicus to the tip of the eighth rib, and below by a line extending from the umbilicus to the tip of the tenth rib. Above this tender area is another area of tenderness which corresponds with another form of visceral disease, and below it is another tender area having its special disease. It is very necessary to observe that these tender areas do not overlap; each is exactly and sharply limited, and, therefore, they do not correspond to nerve roots, for Sherrington has shown that when a nerve root is destroyed the area of anæsthesia overlaps considerably the area of anæsthesia met with when the next nerve root is destroyed. It is also important to note that the whole of a tender cutaneous area is not equally tender, but each area contains one or more points of maximum tenderness, which are always in the same position. A knowledge of the position of these points is very important, for it is to them that a patient always refers his pain. "So fixed are these maxima that supposing a whole area is tender, we can confidently predict that as the disturbance subsides the tenderness will become limited to certain points, and when the disturbance has subsided still more, pain only will be left referred to the same points as the maxima."

Head next turned his attention to cases of herpes zoster, and he points out, what anyone may observe for himself, that the common belief that the eruption follows the course of a nerve or of nerves is incorrect. In the case of the intercostal nerves it nearly follows their distribution, but a cursory examination will always show that the line of the eruption is much more horizontal than that of the nerve. The statement copied from book to book that herpes zoster depends upon inflammation of the posterior nerve roots dates back from more than thirty years ago, when it was originally made by Von Bärensprung, and as it has never been confirmed we may conclude that it is incorrect. Head, however, distinctly proves that the areas affected by herpes zoster exactly correspond to the tender areas which we have just described, and, like them, never overlap. "Moreover, the advent of the eruptions is mostly preceded by pain and tenderness of the skin. In three cases in which I was fortunate enough to see the patient before the eruption developed, I was able to mark out an area on the skin which exactly corresponded to that seen in visceral disease." He was also able to show that the herpetic eruption always appeared first at certain points, and these points correspond with the points of maximum tenderness just described. "Thus the areas occupied by a herpetic eruption correspond to those which become tender in visceral disturbance in three points. First, they have the same distribution; second, they do not overlap; third, they have the same maxima." Then there follows a careful account of each tender area, in which it is stated what visceral disease corresponds to it, and cases of herpes zoster illustrating the extent are given. In all, nineteen areas are described, extending over the trunk and limbs; those of the head and neck are left for a subsequent paper.

As these areas do not correspond to spinal nerve roots, the next problem to be solved was to find out their significance. It is extremely unlikely that they bear any relationship to cutaneous areas, and they can easily be shown not to correspond to peripheral nerves; therefore, by a process of exclusion they must correspond to spinal segments. The first argument in proof of this is that while the limits of an analgesic area are ill-defined when a spinal root is affected, they are well marked and definite, as are Head's areas of tenderness, when the analgesia is due to a lesion of the spinal cord itself. Head gives a series of cases of injury to the spinal cord which most clearly bring out the close relationship between areas of analgesia produced by such injuries and the areas which are tender in visceral disease. In passing, it is well to note that in comparing the areas in the two cases it is important only to have regard to painful stimuli, for in cases of lesion of the spinal cord the area of loss of sensation of pain is definitely marked, while the limits of the area of loss of common sensation are indefinite. As the analgesic area may be found to be affected with loss of sensation to heat and cold, and as it is liable to trophic eruptions, we may conclude that the various spinal segments which minister to the function of perceiving pain and differences in temperature and which preside over nutrition do not overlap, but that the representation of touch in the spinal cord is not confined to definite segments.

If the reader has followed this *résumé* up to the present point he will perceive the great importance of the conclusion that, inasmuch as in disease of any given viscus the same area of skin is always tender, and inasmuch as when in a localised spinal cord lesion precisely the same area is affected with loss of sensation to pain, heat, and cold, and is liable to trophic disturbances, we may conclude that the viscus and the tender area of skin are supplied by the same spinal segment. But as the localised lesion of the cord gives us the precise position of the spinal segment, we actually know which segments of the spinal cord correspond to the different viscera, and Head has been able to map out the spinal cord into a number of segments, each of which corresponds to a certain viscus and to a certain area of skin. Of course a large viscus as the lung may have more than one area corresponding to it. This is an immense advance in our anatomical and physiological knowledge of the spinal cord, but Head's brilliant researches have this additional advantage over many noteworthy anatomical and physiological advances, namely, that the clinical utility is at once obvious and far-reaching, for it is of very great value to the clinical physician to be able to confidently predict that because this particular area of skin is tender that particular viscus must be diseased. Head has also greatly advanced our knowledge of herpes, and has shown that it corresponds to the spinal segments, and he has also taught us the frequency of cutaneous tenderness in visceral disease. In our next article we propose to examine those of his cases in which there was a detailed examination, in order to try and learn exactly what areas of cutaneous tenderness correspond to particular viscera.

